# Effects of 12-Week Progressive Sandbag Exercise Training on Glycemic Control and Muscle Strength in Patients with Type 2 Diabetes Mellitus Combined with Possible Sarcopenia

**DOI:** 10.3390/ijerph192215009

**Published:** 2022-11-15

**Authors:** Yu-Hsuan Chien, Chia-Jen Tsai, Dean-Chuan Wang, Pin-Hung Chuang, Hwai-Ting Lin

**Affiliations:** 1 Division of Endocrinology and Metabolism, Department of Internal Medicine, Kaohsiung Chang Gung Memorial Hospital, College of Medicine, Chang Gung University, Kaohsiung 833, Taiwan; 2 Department of Sports Medicine, Kaohsiung Medical University, Kaohsiung 807, Taiwan; 3 Department of Medical Research, Kaohsiung Medical University Hospital, Kaohsiung 807, Taiwan; 4 Department of Leisure and Sports Management, Cheng Shiu University, Kaohsiung 833, Taiwan

**Keywords:** quality of life, low-resistance, high repetition, physical fitness, blood sugar

## Abstract

Patients with type 2 diabetes mellitus (T2DM) are at a three-fold increased risk of developing sarcopenia compared to those without diabetes. The objective of this study was to investigate whether an intervention involving progressive sandbag exercises is beneficial to patients with T2DM and possible sarcopenia in terms of enhancing muscle strength and controlling blood sugar levels. Forty patients with T2DM and possible sarcopenia (age > 50 years) were recruited and randomly divided into resistance training and control groups. Resistance exercises for the upper and lower extremities were performed using sandbags (0.5 kg at the beginning to 1 kg after 1 month). Patients in the control group were asked to maintain their usual daily lifestyle. After 12 weeks, the training group were significant better than the control group in terms of glycosylated hemoglobin, the five times sit-to-stand test, skeletal muscle mass and calf circumference, and the physiological domain of the World Health Organization Quality of Life Questionnaire. In conclusion, these simple home exercises are beneficial to patients with T2DM combined with possible sarcopenia. This approach can assist patients in controlling their levels of glycosylated hemoglobin as well as improve physical fitness and quality of life.

## 1. Introduction

Type 2 diabetes mellitus (T2DM) is a chronic metabolic disease characterized by hyperglycemia. It accounts for 90% of diabetes cases, and is one of the most common endocrine and metabolic diseases. According to data from the International Diabetes Federation, the number of patients with diabetes worldwide reached 537 million in 2021; it is estimated that this number will increase to 783 million by 2045 [1]. The main cause of T2DM is insufficient β-cell secretion or tissue insulin resistance (IR). It has been proposed that IR of skeletal muscle is the first metabolic defect in patients with T2DM. Accounting for 70–80% of insulin-mediated glucose uptake in the skeletal muscles when blood sugar levels are normal and >90% under hyperglycemia [2]. According to currently available evidence, mechanisms related to T2DM include the accumulation of glycation end products caused by IR, inflammatory response, and hyperglycemia [3]. These effects result in deterioration of muscle strength and muscle mass in patients with diabetes and lead to the development of sarcopenia. The rate of sarcopenia is three-fold higher in patients with T2DM than in individuals without diabetes [4].

The more physical activity a patient undertakes, the more effective this is in preventing the occurrence of T2DM with sarcopenia [5]. According to the exercise guidelines established by the American Diabetes Association, patients with diabetes should perform at least 150 min of aerobic exercise and resistance training at least two to three times per week, and follow the principle of progressive resistance training [6]. Previous studies have shown that aerobic exercise was more effective than resistance exercise in controlling blood sugar [7,8]; however, other studies demonstrated that resistance training was also beneficial for the control of blood sugar levels in patients with T2DM [9,10]. In addition, the quality of life of patients with diabetes is lower than that of healthy individuals due to the psychological impact of blood sugar control or complications [11]. Moreover, the muscle strength and muscle mass loss of patients may reduce physical activity [12], and it also increases the risk of falls and hospitalization and reduces the quality of life.

Previous studies have shown that high-intensity resistance training (~80% one-repetition maximum) increases muscle strength [13,14]. However, if safety is the main consideration, high-repetition/low-resistance training is better than high-resistance/low-repetition training; the former type of exercise is more feasible for elderly patients than the latter type. In some studies, progressive low-resistance training also exerted a similar beneficial effect on muscle strength [15]. Owing to the COVID-19 pandemic, the availability of effective home rehabilitation exercises is crucial for patients with T2DM combined with sarcopenia. Elastic bands are often used as home resistance training instruments. They have been utilized by elderly patients [16] and those with sarcopenia and diabetes to improve muscle strength [17]. However, it is difficult to quantify the training intensity performed by a person using elastic bands, and this type of training is therefore associated with more inconsistent results [16,17]. Moreover, as elastic bands need to be stretched by the user’s strength, there is a certain skill requirement during training. Wearing sandbags on the extremities has been used by patients with stroke in treadmill training to improve gait, thereby improving balance. The purpose of the present study was to investigate whether wearing sandbags as resistance when performing a home-based exercise program may benefit patients with T2DM combined with possible sarcopenia in terms of glycemic control (glycated hemoglobin A1c [HbA1c]), and also improve their muscle strength (grip strength), physical function (five-time chair stand test), skeletal muscle mass and calf circumference, and quality of life.

## 2. Materials and Methods

### 2.1. Study Design and Setting

This prospective, randomized, controlled trial was reviewed and approved by the Institutional Review Board of Kaohsiung Chang Gung Memorial Hospital (202001276B0) and registered with ClinicalTrials.gov (NCT04647617). Patients were recruited from the cases of the Pay-for-Performance Program for Diabetes under National Health Insurance in Taiwan and treated as outpatients in the Division of Endocrinology and Metabolism of Kaohsiung Chang Gung Memorial Hospital. The clinical staff took the initiative to approach patients who met the inclusion criteria during routine health education. Written informed consent was obtained from eligible patients after explaining the purpose of the study. Subsequently, the serial number of each patient’s visit was used to randomly assign them to either the resistance training (odd number) or control group (even number). Muscle strength, physical performance, HbA1c levels, blood fat parameters, muscle mass, calf circumference, and quality of life of all enrolled patients were examined before and after 12 weeks of intervention. The parameters of HbA1c and blood fat were measured at the Kaohsiung Chang Gung Memorial Hospital Laboratory; all other data measurements were conducted by a single researcher in the outpatient clinic of the Endocrinology and Metabolism Department.

### 2.2. Patients

The inclusion criteria of this study were: (1) diagnosis of T2DM by endocrinologists and metabolism physicians according to the disease diagnosis code (ICD-10: E110) for patients with T2DM; (2) age > 50 years; and (3) meeting the detection criteria for possible sarcopenia proposed by the 2019 Asian Working Group for Sarcopenia (including calf circumference of <34 cm and <33 cm at the largest circle of respectively male and female calves; and grip strength of <28 kg and <18 kg for males and females, respectively).

The exclusion criteria were as follows: peripheral neuropathy; retinopathy; stroke; diabetic foot or amputation; history of myocardial infarction; advanced renal disease; malignant hypertension; or a history of falls and unstable gait. Throughout the study, prescription drugs used by the patients were not adjusted.

### 2.3. Intervention

#### 2.3.1. Resistance Training Group

**Figure 1:**
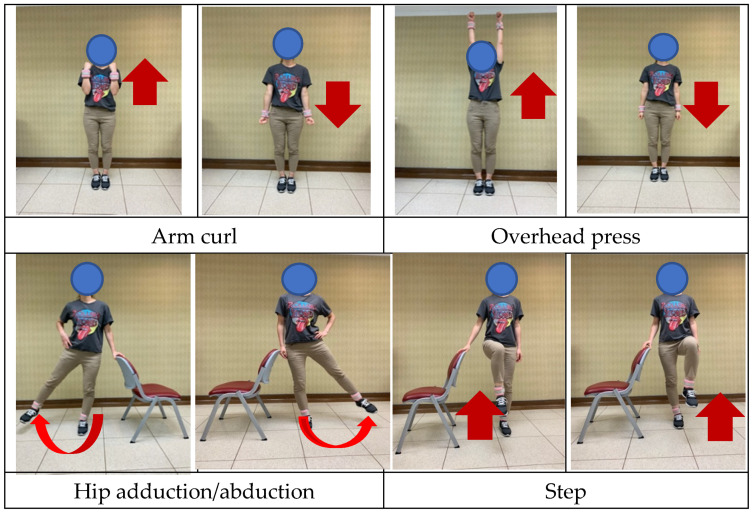

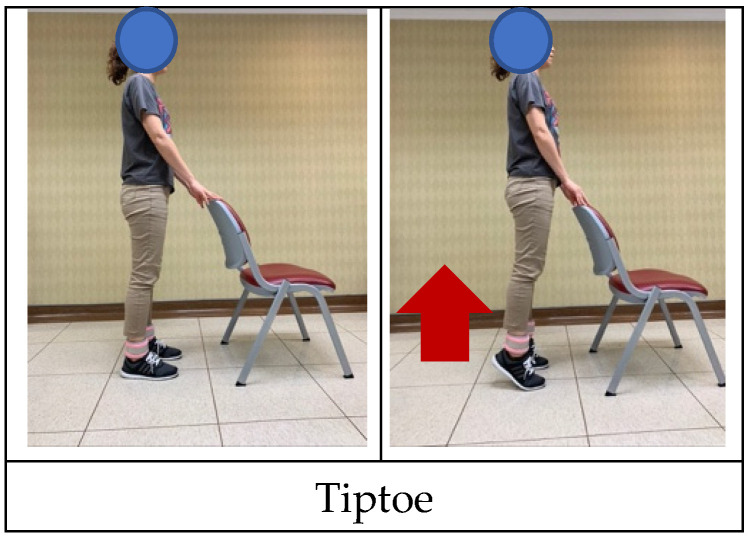
The five movements performed during resistance training.

To accommodate the elderly patients in this study, we intended to design an easy and safe home resistance training exercise. The training muscles are mainly the functional muscles of the limbs. Sandbags were utilized as the resistance training equipment. The patients were instructed on the exercises by the clinical staff during a visit to the outpatient department. The exercises consisted of arm curl (elbow flexion/extension), overhead press (shoulder), hip adduction/abduction, step (hip and knee flexion/extension), and tiptoe (ankle flexion/extension) (Figure 1). Initially, a sandbag weighing 0.5 kg was worn, and each exercise was performed 8–15 times, reaching a rated perceived exertion (RPE) of 13 (range: 6–20), which was perceived as somewhat hard. The training exercises included 5–10 min of warm-up and cool-down before and after the main exercise to prevent muscle soreness and injury. The main exercise consisted of three sets with 1–2 min of rest between sets; the total duration of each exercise was approximately 30 min. Based on the recommendation in the guidelines of the American College of Sports Medicine, patients should perform strength training exercises three times weekly. Participants’ adherence to the exercise program was tracked by phone call or social software once weekly. Exercises were intensified by adding repetitions of each movement until the patients could easily perform 20 repetitions. Subsequently, the weight of the sandbags was changed to 1 kg. To ensure the quality and safety of exercises at home, patients and their families were required to test whether all required movements could be performed correctly in the education room. Each participant also received a handout with demonstration photos as a reminder.

#### 2.3.2. Control Group

Patients in the control group were asked to maintain their usual daily lifestyle as much as possible.

### 2.4. Outcome Measures

#### 2.4.1. Biochemical Analysis

Blood glucose levels are representative of the short-term blood sugar control situation, and the levels of HbA1c in the blood reflect the blood sugar control status during a period of 3 months [18]. The normal levels of HbA1c range between 4% and 5.6%; levels > 6.5% are indicative of diabetes. In this study, the levels of HbA1c were measured using a high-performance liquid chromatography (HPLC) system (Premier Hb9210; Bray, Kansas City, MO, USA).

Blood lipids, mainly including triglyceride and total cholesterol, are insoluble lipids, mainly originating from dietary patterns and liver synthesis and also tested in this study. Total cholesterol is divided into low-density lipoprotein (LDL) and high-density lipoprotein (HDL).

#### 2.4.2. Grip Strength

Grip strength is generally regarded as the most convenient parameter for assessing muscle strength. It is also an indicator used for the diagnosis of sarcopenia. In the present study, an electronic digital grip strength dynamometer (TKK; Takei Scientific Instruments, Tokyo, Japan) was used for testing. First the width of the grip was adjusted. The ideal width is an angle of 90° between the second knuckle of the index finger and the grip. The patient then adopted a standing posture with the feet naturally separated, the body maintained upright, and the arms naturally relaxed. The patients were asked to practice the exercise first and then use the dynamometer to press forcefully and steadily for 5 s. The measurement was performed twice, with a 1 min interval between the two recordings, and the average value was calculated.

#### 2.4.3. Five Times Sit-to-Stand Test

Diagnosis of sarcopenia requires concurrent assessment of the quality and quantity of the muscles of elderly individuals (i.e., muscle mass, muscle strength, physical performance, etc.). The five times sit-to-stand test is suitable for this purpose. Briefly, the patient sat on a chair with a height of approximately 43 cm without armrests. The patient then stood up from the chair and then sat down again, carrying out five repetitions and timing the total process with a stopwatch. Patients were asked to perform the exercise according to their physical condition.

#### 2.4.4. Muscle Mass

A body composition analyzer (Tanita BC545N; Tanita Corporation, Tokyo, Japan) was used to measure the appendicular skeletal muscle mass (ASM) and determine the appendicular skeletal muscle mass index (ASMI). Before the test, the patients were asked to empty their bladder and enter their basic information, including height, weight, age, gender and other information, on the analyzer screen. Alcohol (75%) was used to wipe clean the palm surface of the patients and electrodes of the analyzer.

#### 2.4.5. Calf Circumference

Calf circumference is often used to roughly assess the amount of muscle mass. Males and females with calf circumference measuring <34 cm and <33 cm, respectively, are at high risk of developing sarcopenia. Patients were asked to take a standing position, and the largest diameter of the right calf was measured with a measuring tape.

#### 2.4.6. WHOQOL-BREF Taiwan Version

The patients were asked to fill in their own quality of life status for the previous 2 weeks in the World Health Organization Quality of Life Questionnaire brief version (WHOQOL-BREF). The scoring of this questionnaire is based on a Likert five-point scale (scores of five and one indicating the highest and lowest points, respectively). The score was calculated by determining the average value for each question in the same domain and multiplying the value by four. The total score (based on the formula) was converted to a range of 0–20 points, with higher scores indicating better quality of life.

### 2.5. Statistical Analysis

A priori sample size estimation was based on anticipated differences in grip strength of the patients with possible sarcopenia as the primary outcome based on an estimated large effect size (0.8) between the pretest and post-training measurements [19]. The calculation was also based on an alpha level of 0.05 and a desired statistical power of 80% using G*Power. The estimated minimum sample size was 18 patients per group. Assuming a dropout rate of 10%, we thus enrolled 20 patients in each group.

Statistical analysis was performed using the SPSS statistical software package (version 20.0; IBM Corp, Armonk, NY, USA). All data values are represented as the mean and standard deviation. The Shapiro–Wilk method was used to test whether the data were normally distributed, and the homogeneity of the two groups was confirmed by Levene’s test. The chi-squared test was used to analyze differences in gender and drug use between the two groups. Generalized estimating equations (GEE) were used to test the main effect of time and group, and a significant group × time interaction. The paired *t*-test was used to examine the differences in every testing parameter before and after training (time) in each group. The significance level was set at *p* < 0.05.

## 3. Results

### 3.1. Flow of Participants

**Figure 2:**
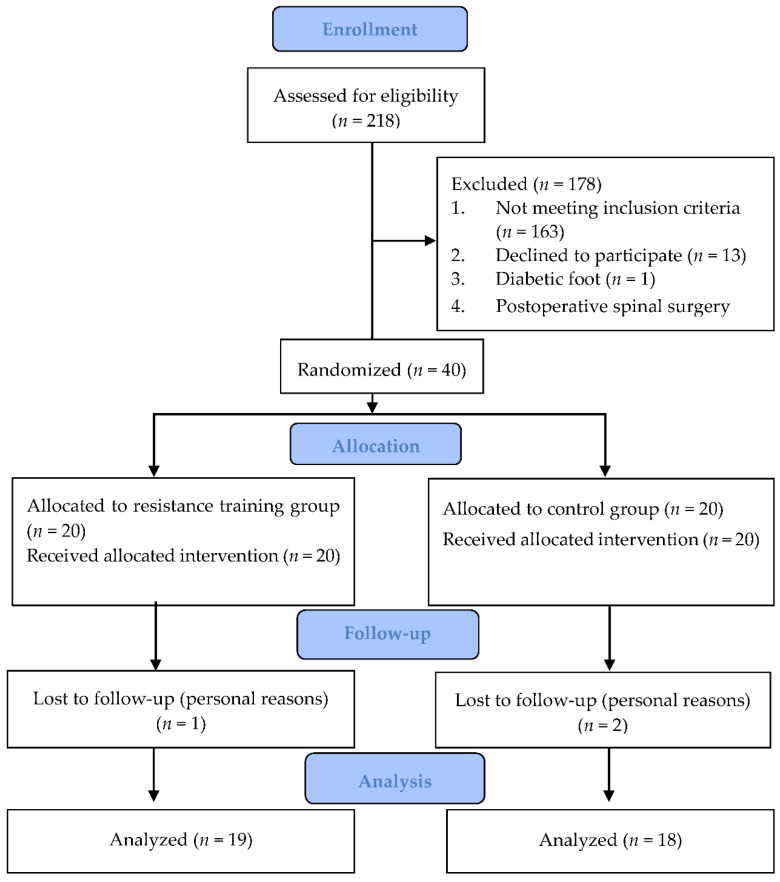
CONSORT flow diagram.

A total of 218 patients diagnosed with T2DM with possible sarcopenia were recruited for the study. Of these, 178 patients who did not meet the inclusion criteria were excluded. The remaining 40 eligible patients were randomly assigned to the resistance training and control groups. Three patients were excluded later due to personal reasons or missed visits to the hospital. Finally, 37 patients completed the entire experiment (19 and 18 in the resistance training and control groups, respectively) (Figure 2). The adherence rate of patients who participated in the intervention in the training group was 82%. There were no significant differences in demographic characteristics, history of diabetes, HbA1c, blood fat, and medication use between the two groups (*p* > 0.05) (Table 1). In addition, none of the participants experienced exercise injury during the intervention.

### 3.2. Effect of Intervention on HbA1c

For the HbA1c levels, the main effects changed significantly over time within (*p* = 0.037) and between the groups (*p* = 0.042). A significant interaction between time and group (*p* = 0.004) was also observed. After 12 weeks of intervention, the HbA1c levels were decreased significantly in both groups (*p* < 0.001 (training); *p* = 0.045 (control)); nevertheless, the training group showed a more significant improvement in HbA1c than the control group (Table 2).

### 3.3. Effect of Intervention on the Lipid Profile

For the HDL cholesterol, LDL cholesterol, and triglyceride levels, no statistical significance was reached for the main effects over time within groups (*p* = 0.416, 0.272, and 0.669, respectively), between groups (*p* = 0.078, 0.370, and 0.595, respectively), or in time-by-group interaction (*p* = 0.354, 0.072, and 0.172, respectively) (Table 2).

### 3.4. Effect of Intervention on Grip Strength

For the grip strength, the main effects were statistically significant over time within the group (*p* = 0.001). The grip strengths were decreased significantly in both groups (*p* < 0.001(training); *p* = 0.021(control)). There were no significant differences found between groups (*p* = 0.520) or for the time-by-group interaction (*p* = 0.097) (Table 2).

### 3.5. Effect of Intervention on the Five Times Sit-to-Stand Test

Performance in the five times sit-to-stand test showed a statistically significant effect in time-by-group interaction (*p* = 0.001). However, statistical significance was not reached for the main effects over time within (*p* = 0.061) or between (*p* = 0.499) groups. The test time for the training group decreased with time, whereas that for the control group tended to increase (Table 2).

### 3.6. Effect of Intervention on ASM and ASMI

For ASM and ASMI, the main effects were statistically significant over time within groups (*p* = 0.009 and 0.041, respectively) and for the time-by-group interaction (*p* = 0.001 and 0.001, respectively). However, statistical significance was not reached for the main effects over time between groups (*p* = 0.795 and 0.235, respectively). After 12 weeks, the ASM and ASMI increased over time in the training group (*p* = 0.023 and 0.033, respectively) but decreased in the control group (*p* = 0.022 and 0.025, respectively) (Table 2).

### 3.7. Effect of Intervention on Calf Circumference

For the calf circumference, there was a statistically significant effect in terms of time-by-group interaction (*p* = 0.010). However, statistical significance was not reached for the main effects over time within (*p* = 108) or between (*p* = 0.223) groups. The calf circumference in the training group increased with time (*p* < 0.001), while that in the control group tended to decrease (*p* = 0.069) (Table 2).

### 3.8. Effect of Intervention on the WHOQOL Scale

Scoring on the WHOQOL scale in the physical domain showed a statistically significant effect in terms of time-by-group interaction (*p* = 0.090) (Table 3). However, statistical significance was not reached for the main effects over time within (*p* = 0.921) or between (*p* = 0.371) groups. In the general health, psychological, environmental, and social relationships domains, statistical significance was not reached for the main effects over time within groups (*p* = 0.953, 0.509, 0.787, and 0.927, respectively) or between groups (*p* = 0.703, 0.957, 0.582, and 0.631, respectively), or time-by-group interaction (*p* = 0.304, 0.354, 0.540, and 0.167, respectively). Twelve weeks of training resulted in an increase in physical domain scores (*p* = 0.003); however, the intervention did not lead to significant change in the control group (*p* = 0.222).

## 4. Discussion

To our knowledge, this is the first prospective study on the effect of low-intensity resistance exercise training at home in patients with T2DM combined with possible sarcopenia. Compared with patients in the control group, those in the training group had more significantly improved blood biochemical results (HbA1c) and better conditions (time*group interaction) in muscle strength and physical performance (five times sit-to-stand test), limb muscle mass, calf circumference, and quality of life. Exercise compliance in the training group was 82% throughout the experimental period and was higher than reported in previous studies [20].

Previous investigations have shown that aerobic exercise was more effective than resistance exercise in controlling blood sugar [7,8]. Currently, few studies have examined the effects of intervention with progressive low-weight-bearing resistance exercise in patients with T2DM. These studies reported a decrease in the average HbA1c levels by 0.3–0.5% [10,21,22]; these results are similar to the present findings. Currently, the mechanism through which patients with diabetes can achieve blood sugar control through resistance exercise remains unclear. However, it is likely that the increase of glucose transporter type 4 (GLUT4) enhances the ability of insulin transport and improves IR [23].

Consistent with previous results, the present study did not demonstrate statistically significant differences in terms of time and group effects for HDL, LDL, and triglycerides, [7,22]. Previous studies suggested that weekly exercise must exceed a caloric cost of 1200–2200 kcal to exert a significant effect on blood lipids; in the present study, the limited exercise may be the main reason for the lack of effect [24,25,26]. Moreover, 95% of the patients in this study were receiving treatment with blood lipid-lowering drugs. Under this condition, the HDL and LDL levels of the two groups prior to this study had reached the standard value recommended by the American Diabetes Association (HDL ≥ 50 mg/dL, LDL < 100 mg/dL, triglycerides < 150 mg/dL); this may be the cause for the observed limited improvement in cholesterol levels after training.

For older adults, resistance training can reverse the negative effects of aging and is key to maintaining strength. The re-recruitment of muscle fibers through training increases their volume to generate more force [27]. Studies have shown that, in older adults who have never received previous resistance training, both skeletal muscles and neuromuscular systems exhibit significant adaptability and plasticity in response to strength training [28]. None of the patients recruited in the present study had received resistance training prior to their participation. After 12 weeks of sandbag training, the grip strength of patients increased by 3.2 kg, and the required five times sit-to-stand test duration was shortened by 3.2 s. Therefore, based on the results of this study, resistance training with low intensity may increase muscle strength and physical performance in the elderly [29,30,31,32].

A Cochrane meta-analysis concluded that progressive resistance training is an effective interventional strategy for improving physical performance in older adults, with benefits including increased muscle strength and muscle mass [33]. In this study, 12 weeks of progressive sandbag resistance training was applied. Although the load was relatively light, the skeletal muscle mass of the limbs in the training group increased by 0.3 kg, which was also similar to the results of a previous study [17,19,23,34]. It can thus be concluded that the cross-sectional area of type II muscle fibers can be increased through low-intensity and high-repetition exercise [35]. The calf circumference of the training group in this study increased from 30.4 cm to 31.1 cm (an increase of 0.7 cm). According to a study conducted by Yang, low resistance training exerted a positive effect on calf circumference and limb muscle mass [36]. Nonetheless, studies conducted in Japan and Korea yielded different results, which may be due to differences in gender, environment, ethnicity, genetics, lifestyle, diet, iliac structure, fat distribution, and skin wrinkling due to disease [37,38].

The pathogenesis of T2DM increases the rate of muscle strength and muscle mass loss, resulting in a decrease in physical activity. It has been shown that resistance training improves quality of life, such as overall mood, sleep quality, increased vitality, and self-efficacy. Moreover, Pourgathi showed that resistance training improved the quality of life of elderly individuals through an increase in muscle strength and muscle mass [16]. Therefore, the elderly can be encouraged to add progressive resistance exercise to their usual exercise according to their ability and maintain their daily independence [12,34,35]. However, we found no significant difference before and after tests in both groups in the general health, psychological, social relationship, and environmental domains. This lack of effect may be related to the distress, psychological pressure, frustration, and powerlessness caused by diabetes. The results of the quality of life questionnaires for patients with T2DM may also vary greatly depending on the treatment modality, the presence or absence of comorbidities and complications, the length of the disease period, and basic demographic variables.

This study has a number of limitations. Firstly, all included patients were elderly and most resided far away from the hospital, making it difficult for them to frequently return to the hospital for assessment. Therefore, the exercise status of the patients could only be tracked through telephone interviews and communication software. Secondly, exercise compliance may not actually reflect the patient’s exercise status at home. The diet and lifestyle of patients with diabetes have a great impact on physiological parameters, such as blood sugar and lipid levels, but in this study, the diet of the patients was not controlled. It is suggested that future studies make use of cooperation with dietitians to control the diet of patients. Finally, the measurement of biochemical parameters may be affected by the type of drugs used by the patient (e.g., diuretics, excretion and hypoglycemic drugs, and others), which were not controlled in this study.

## 5. Conclusions

In this study, patients with T2DM and possible sarcopenia (age > 50 years) performed 12 weeks of home-based progressive quantitative sandbag resistance exercise training for progressive upper and lower limb muscle strength using low-load quantitative sandbags. Although all subjects’ medication status did not change after 12 weeks of training, the resistance exercise training improved the levels of HbA1c, upper extremity strength, and muscle mass, and had better sit-to-stand performance and quality of life than the control group.

During this study, there were no safety concerns with regard to exercise (e.g., hypoglycemia, falls). Therefore, this approach can be used for elderly diabetic patients with poor physical fitness to increase muscle strength. The designed exercise methods can be easily performed at home, the equipment is easy to use, telephone interviews or communication software can be utilized to provide continuous care, and the training method is cost-effective. This approach provides a new option for the elderly to exercise at home during the current COVID-19 pandemic.

## Figures and Tables

**Table 1:** Baseline characteristics of patients.

	Training Group (*n* = 20)	Control Group (*n* = 20)	*p-*Value
Gender ^b^			
Male/female, *n* (%)	5 (25%)/15 (75%)	2 (10%)/18 (90%)	0.212
Age (years) ^a^	67.6 ± 7.7	67.3 ± 6.1	0.892
Duration of T2DM (years)	17.5 ± 16.3	13.6 ± 7.6	0.346
Body height (cm) ^a^	154.4 ± 16.3	156.3 ± 6.0	0.349
Body weight (kg)	58.0 ± 9.0	62.4 ± 9.9	0.149
Body index (kg/m^2^)	24.3 ± 3.4	25.5 ± 3.7	0.311
HbA1c (%)	8.1 ± 1.1	7.6 ± 0.6	0.057
LDL (mg/dL) ^a^	86.9 ± 36.5	77.7 ± 29.7	0.388
HDL (mg/dL) ^a^	54.4 ± 13.6	47.5 ± 11.6	0.094
Triglycerides (mg/dL) ^a^	117.4 ± 32.1	125.4 ± 61.2	0.608

T2DM = type 2 diabetes mellitus; LDL **=** low-density lipoprotein; HDL = high-density lipoprotein. Values are presented as the mean ± standard deviation; ^a^: analyzed by the independent *t* test; ^b^: analyzed by the chi-squared test.

**Table 2:** Changes in outcomes between groups after 12 weeks of intervention.

Outcomes	Group	Baseline	Post	Within Group *p-*Value	Between Groups *p-*Value	Time * Group *p-*Value
HbA1c (%)	Training	8.1 ± 1.1	7.7 ± 0.9	0.037 *	0.042 *	0.004 *
	Control	7.6 ± 0.6	7.4 ± 0.7			
HDL (mg/dL)	Training	54.4 ± 13.6	53.7 ± 11.8	0.416	0.078	0.354
	Control	47.5 ± 11.6	48.8 ± 12.6			
LDL (mg/dL)	Training	87.0 ± 36.5	76.6 ± 27.0	0.272	0.370	0.072
	Control	77.8 ± 29.7	82.7 ± 31.2			
TG (mg/dL)	Training	117.4 ± 32.1	125.4 ± 61.2	0.669	0.595	0.172
	Control	100.3 ± 36.2	130.9 ± 65.0			
Hand grip (kg)	Training	15.9 ± 5.0	19.1 ± 4.9	0.010 *	0.520	0.097
	Control	15.1 ± 3.8	16.5 ± 5.4			
Five times sit-to-stand test (s)	Training	16.6 ± 3.5	13.4 ± 3.2	0.061	0.499	0.001 *
	Control	15.9 ± 3.0	17.1 ± 4.7			
ASM (kg)	Training	16.7 ± 3.3	17.1 ± 3.5	0.009 *	0.795	0.001 *
	Control	16.9 ± 2.1	16.6 ± 2.2			
ASMI (kg/m^2^)	Training	6.9 ± 0.9	7.1 ± 1.0	0.009 *	0.795	0.001 *
	Control	6.9 ± 0.7	6.8 ± 0.1			
Calf circumference (cm)	Training	30.4 ± 2.3	31.2 ± 1.9	0.009 *	0.795	0.001 *
	Control	31.3 ± 2.0	30.6 ± 2.8			

ASM = appendicular skeletal muscle mass; ASMI = appendicular skeletal muscle mass index; HDL = high-density lipoprotein; LDL = low-density lipoprotein; TG = triglycerides; * Significant difference (*p* < 0.05).

**Table 3:** Changes in WHOQOL scale between groups after 12 weeks of intervention.

Domain	Group	Baseline	After 12 Weeks	Within Group *p-*Value	Between Groups *p-*Value	Time * Group *p-*Value
General health	Training	2.8 ± 0.6	3.0 ± 0.6	0.953	0.703	0.304
	Control	2.7 ± 0.6	2.7 ± 0.8			
Physical domain	Training	10.6 ± 1.8	12.1 ± 1.8	0.921	0.371	0.009 *
	Control	11.1 ± 1.7	11.1 ± 2.3			
Psychology domain	Training	10.8 ± 1.8	11.4 ± 1.8	0.509	0.957	0.354
	Control	10.8 ± 1.8	10.4 ± 2.4			
Social relationship domain	Training	11.7 ± 1.4	11.9 ± 1.4	0.787	0.582	0.540
	Control	11.4 ± 2.0	11.4 ± 2.1			
Environmental domain	Training	14.0 ± 2.0	14.5 ± 1.7	0.927	0.631	0.167
	Control	14.3 ± 1.7	14.3 ± 1.8			

WHOQOL = World Health Organization Quality of Life. * Significant difference (*p* < 0.05).

## Data Availability

Data can be made available upon request.
